# A phase III, multicenter, randomized controlled trial of preoperative versus postoperative stereotactic radiosurgery for patients with surgically resectable brain metastases

**DOI:** 10.1186/s12885-022-10480-z

**Published:** 2022-12-30

**Authors:** Subhadip Das, Salman Faruqi, Robert Nordal, Yves Starreveld, John Kelly, Gregory Bowden, John Amanie, Alysa Fairchild, Gerald Lim, Shaun Loewen, Lindsay Rowe, Carla Wallace, Sunita Ghosh, Samir Patel

**Affiliations:** 1 Division of Radiation Oncology, Department of Oncology, Tom Baker Cancer Center, Calgary, Alberta Canada; 2 Division of Neurosurgery, Department of Clinical Neurosciences, University of Calgary, Calgary, Alberta Canada; 3 Division of Neurosurgery, Department of Surgery, University of Alberta, Edmonton, Alberta Canada; 4 Division of Radiation Oncology, Department of Oncology, University of Alberta, Cross Cancer Institute, 11560 University Avenue, Edmonton, AB T6G 1Z2 Canada; 5 Department of Radiology, University of Calgary, Calgary, Alberta Canada; 6 Division of Medical Oncology, Department of Oncology, University of Alberta, Edmonton, Alberta Canada

**Keywords:** Brain metastases, Stereotactic radiosurgery, Preoperative, Postoperative, Quality of life, Neurocognitive outcomes

## Abstract

**Background:**

Postoperative stereotactic radiosurgery (SRS) is a standard management option for patients with resected brain metastases. Preoperative SRS may have certain advantages compared to postoperative SRS, including less uncertainty in delineation of the intact tumor compared to the postoperative resection cavity, reduced rate of leptomeningeal dissemination postoperatively, and a lower risk of radiation necrosis. The recently published ASCO-SNO-ASTRO consensus statement provides no recommendation for the preferred sequencing of radiotherapy and surgery for patients receiving both treatments for their brain metastases.

**Methods:**

This multicenter, randomized controlled trial aims to recruit 88 patients with resectable brain metastases over an estimated three-year period. Patients with ten or fewer brain metastases with at least one resectable, fulfilling inclusion criteria will be randomized to postoperative SRS (standard arm) or preoperative SRS (investigational arm) in a 1:1 ratio. Randomization will be stratified by age (< 60 versus ≥60 years), histology (melanoma/renal cell carcinoma/sarcoma versus other), and number of metastases (one versus 2–10). In the standard arm, postoperative SRS will be delivered within 3 weeks of surgery, and all unresected metastases will receive primary SRS. In the investigational arm, enrolled patients will receive SRS of all brain metastases followed by surgery of resectable metastases within one week of SRS. In either arm, single fraction or hypofractionated SRS in three or five fractions is permitted. The primary endpoint is to assess local control at 12 months in both arms. Secondary endpoints include local control at other time points, regional/distant brain recurrence rates, leptomeningeal recurrence rates, overall survival, neurocognitive outcomes, and adverse radiation events including radiation necrosis rates in both arms.

**Discussion:**

This trial addresses the unanswered question of the optimal sequencing of surgery and SRS in the management of patients with resectable brain metastases. No randomized data comparing preoperative and postoperative SRS for patients with brain metastases has been published to date.

**Trial registration:**

Clinicaltrials.gov, NCT04474925; registered on July 17, 2020. Protocol version 1.0 (January 31, 2020). Sponsor: Alberta Health Services, Edmonton, Canada (Samir Patel, MD).

## Background

Brain metastases occur in 10–30% of cancer patients and can lead to devastating neurological deficits and neurocognitive decline that can significantly affect quality-of-life [1–3]. Surgical resection of intracranial metastases can be indicated for patients with larger tumors (> 2 cm in diameter), symptomatic lesions, or need for tissue for diagnosis or clinical trial enrollment [4]. Postoperative whole brain radiotherapy (WBRT) has been shown to improve local control and reduce neurological death rate after intracranial tumor resection [5, 6]. Concerns about neurocognitive decline resulting from WBRT led to a multicenter randomized phase III trial by Brown et al. comparing postoperative WBRT with stereotactic radiosurgery (SRS) in 194 patients. With a median follow up of 11.1 months, cognitive function and quality of life were better in SRS arm without any significant difference in overall survival [7]. Postoperative SRS is now considered a standard of care option for patients in addition to WBRT.

Treatment planning tends to be more complicated with postoperative SRS than in the preoperative setting, and this factor has been suggested to contribute to the higher local failure rate seen in the SRS arm compared to WBRT in trial by Brown et al. [7]. Further, leptomeningeal disease (LMD) has frequently been reported in case series of postoperative SRS [8–13]. Preoperative SRS has been suggested to help ease target delineation to simplify treatment planning, reduce the planning target volume (PTV) margin to reduce the treatment volume, shorten the overall treatment time, and reduce the risk of leptomeningeal dissemination after surgery [14].

Patel et al. published findings from a multi-institutional retrospective study comparing preoperative and postoperative SRS in 180 patients [15]. The median follow-up period was 24.6 months, and 36.7% of all patients received preoperative SRS. There was no significant difference between the two groups in terms of local recurrence, overall survival, and distant brain recurrence. However, postoperative SRS was associated with higher rates of leptomeningeal recurrence and symptomatic radiation necrosis (SRN). The SRN rate was higher in postoperative patients (16.4%) compared to preoperative patients (4.8%) at two years. The LMD rate was 16.6% in postoperative patients compared to 3.2% of preoperative patients (*p* = 0.010). The authors suggested that sterilization of the treatment field by preoperative SRS may restrict tumor cell dissemination during surgery to account for the lower rate of LMD with preoperative SRS. The PROPS-BM multicenter, retrospective study included 242 patients treated with preoperative SRS [16]. The 2-year rates of LMD and ARE were low at 7.6 and 6.8%, respectively. On multivariate analysis subtotal resection (STR) resulted in worsened local control and overall survival (OS). The authors suggest that a fractionated regimen of radiosurgery for patients at high risk of subtotal resection to increase the biological effective dose delivered to the target volume, consistent with previous literature in patients treated with postoperative SRS [17].

A potential rational for lower SRN in patients treated with preoperative SRS that normal tissue surrounding the SRS target receiving high doses of radiation will subsequently resected thereby decreasing the available injured tissue and cytokine concentration which might augment a radiation necrosis reaction [18, 19]. A contributing factor is that studies of preoperative SRS have used smaller PTV margins, typically 0–1 mm, compared to trials of postoperative SRS (e.g., 2 mm in the NCCTG N107C/CEC.3 trial) [7]. Postoperative contouring guidelines recommend an additional margin of 5–10 mm along the dura if dural contact was observed on preoperative imaging [20]. These rationales are supported by dosimetric data reported by El Sahfie et al. that show reduction in the dose exposure of the normal healthy brain with preoperative versus postoperative SRS [21]. The median volume of healthy brain receiving 28 Gy was 6.79 cc with preoperative SRS compared to 10.79 cc with postoperative SRS (*p* < 0.001).

The ASCO-SNO-ASTRO consensus statement for the treatment of brain metastases with radiosurgery indicates that there is no recommendation regarding the sequencing of surgery and SRS [22]. There are no randomized data comparing preoperative and postoperative SRS currently in the literature [23]. The present study is a prospective, multicenter, randomized, controlled trial comparing preoperative to postoperative SRS in the treatment of resectable brain metastases. Importantly, fractionated SRS for both preoperative and postoperative cases, depending on their size, is allowed in this study. The primary objective of the study is to compare the local control of pre- versus postoperative SRS. Secondary objectives are to compare distant brain recurrence, leptomeningeal recurrence, and overall survival of the two arms as well as the comparison of quality-of-life and neurocognitive outcomes of preoperative versus postoperative SRS (Table 1).

**Table 1:** Study endpoints

**1.0 Primary endpoints** 1.1 Local control at 12 months.	
**2.0 Secondary endpoints** 2.1 Local control at 6 and 24 months 2.2 Distance brain recurrence rate (%) at 6,12, and 24 months 2.3 Leptomeningeal recurrence rate (%) at 6, 12, 24 months 2.4 Overall survival (in months) at 6, 12 and 24 months 2.5 Hopkins Verbal Learning Test Revised score (points) at 3, 6, 9, 12, 16, 24 months 2.6 Controlled Oral Word Association test score (points) at 3, 6, 9, 12, 16, 24 months 2.7 Trail Making Test score (points) at 3, 6, 9, 12, 16, 24 months	

## Methods

### Study design

**Fig. 1:**
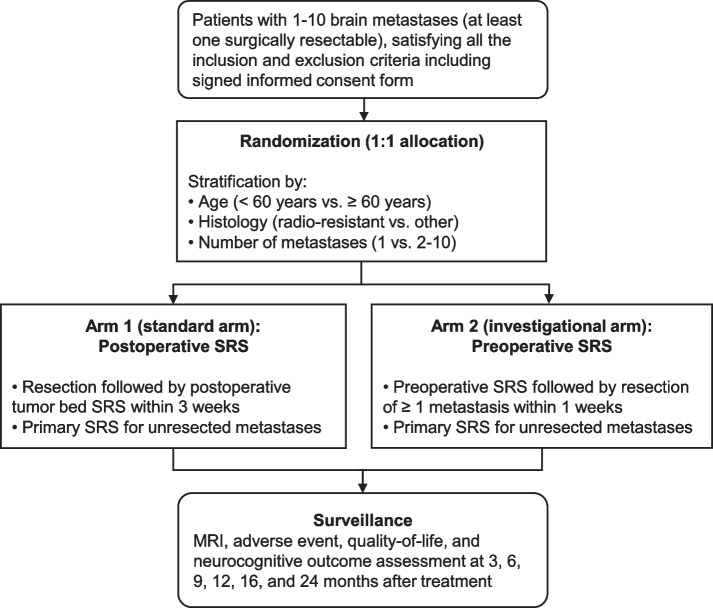
Study schema

This phase III, multicenter, randomized controlled trial will enroll patients brain metastases, of which at least one is resectable. Patients will be randomly assigned to either postoperative SRS (standard arm) or preoperative SRS (investigational arm) in a 1:1 ratio. The study schema is illustrated in Fig. 1.

### Patient selection and eligibility criteria

Patients with between one and ten brain metastases on contrast-enhanced MRI of the brain will be screened for the study. At least one brain metastases should be deemed surgically resectable after image review or consultation with a neurosurgeon. Further patient eligibility criteria are listed in Table 2. If the patient is eligible for enrolment, a detailed discussion of the study including purpose and potential risks will take place with interested patients. The signed, written, informed consent will be obtained from every patient before trial enrollment.

**Table 2:** Patient eligibility criteria

**1.0 Inclusion criteria**	**2.0 Exclusion criteria**
1.1 Age ≥ 18 years 1.2 Pathologically proven primary malignancy 1.3 ECOG performance score of 0–2 1.4 MRI with contrast demonstrating ten or fewer brain metastases, of which at least one is resectable as deemed by a neurosurgeon 1.5 Ability to complete neurocognitive testing without assistance from family or friends. 1.6 Previous SRS to lesions other than the one being resected is allowed 1.7 Patients with childbearing potential must have a negative urine or serum pregnancy test ≤7 days before enrolment 1.8 Participants capable of giving informed consent, or if appropriate participants having an acceptable individual capable of giving consent	2.1 Patients who have received prior WBRT, or SRS to the lesion being resected at time of study accrual 2.2 Patients unable to undergo MRI scan (e.g., pacemaker) 2.3 Leptomeningeal disease 2.4 Germ cell tumor, small cell lung cancer or hematological primary malignancy

*Abbreviations ECOG* Eastern Cooperative Oncology Group, *SRS* stereotactic radiosurgery, *WBRT* whole brain radiotherapy

### Randomization and treatment allocation

Patients will be allocated by blocked randomization (block size of 16), on a 1:1 basis, to either the standard or investigational arm. Patients will be stratified according to the following factors: age (< 60 years versus ≥60 years), histology (melanoma/renal cell carcinoma/sarcoma versus other) and number of metastases (1 versus 2–10). A computer-generated list has been uploaded into the Electronic Data Capture (EDC) system for randomization. After confirming a participant’s eligibility, the study staff will randomize the patient using the EDC. Following randomization, treatment will be initiated within two weeks. Because of the difference in treatment sequencing (before or after surgery), blinding of trial participants or investigators will not be possible; neuropathologists and neuroradiologists will prospectively review applicable material without knowledge of the random assignment.

### Pre-treatment evaluation

The screening assessments will be done within one week of randomization, performed after having received the participant’s written informed consent. These will include their medical history and demographics, physical examination, Karnofsky performance status, adverse event review, steroid dose assessment, pregnancy testing (for females with childbearing potential), diagnostic MRI scan (within 30 days of randomization), quality-of-life questionnaires and neurocognitive testing. The assessments performed prior to consent as per standard institutional guidelines do not need to be repeated if they fall within the 7-day screening window.

### Interventions

The following procedures will be performed during the treatment phase of the trial:

1. Standard arm: Resection of ≥1 brain metastases followed by postoperative tumor bed SRS within 3 weeks.
2. Investigational arm: Preoperative SRS followed by resection of ≥1 brain metastases within 1 week.

Patients treated on both arms will receive primary SRS for unresected brain metastases.

### Radiotherapy

A linear accelerator (LINAC) using ≥4 megavoltage (MV) photons, with collimation using micro-multi-leaf collimators (5 mm width or less at isocenter) or cones, Gamma Knife or Cyberknife may be used to deliver SRS. All patients will be counselled about thermoplastic mask fixation, CT simulation and immobilization in detail. Localization, simulation, and immobilization will be as per institution standard.

For the tumor bed (standard arm only), the gross tumor volume (GTV) will consist of the tumor bed including residual tumor, if present, on T1-weighted MRI (with gadolinium contrast, unless contraindicated) or CT scan (with contrast, unless contraindicated). The clinical target volume (CTV) will be GTV plus 2 mm and can be cropped for natural barriers to spread (e.g., bone) or structures not at-risk for tumor infiltration. The planning target volume (PTV) will be the CTV plus 0 to 1 mm when using mask-based immobilization (no margin will be added when using frame-based immobilization). For intact metastases on both arms, the GTV will consist of all visible tumor on T1-weighted MRI or CT scan. No margin will be added for CTV. The PTV will be the CTV plus 1 mm when using mask-based immobilization (no margin will be added when using frame-based immobilization). Radiosurgery dose and fractionation is based on the NCCTG N107C/CEC.3 trial [7] with modification (Table 3). Treatment will be delivered on consecutive days, one fraction per day, for fractionated radiotherapy regimens.

**Table 3:** Radiotherapy dose and fractionation

Tumor Bed (standard arm) (prescription is based on CTV volume)	Intact metastases (both arms) (prescription is based on the CTV maximal diameter)
Lesions < 4.2 cc receive 20 Gy	Lesions < 1.0 cm receive 22–24 Gy
Lesions ≥4.2 to < 14.4 cc receive 17 Gy	Lesions ≥1.0 to < 2.0 cm receive 20–22 Gy
Lesions ≥14.4 to < 30 cc receive 27 Gy in three or 30 Gy in 5 fractions	Lesions ≥2.0 to < 3.0 cm receive 18–20 Gy
Lesions ≥30 cc receive 24–27 Gy in 3 fractions or 30 Gy in five fractions	Lesions ≥3.0 to < 5.0 cm receive 27 Gy in 3 or 30 Gy in five fractions
	Lesions ≥5.0 cm receive 24–27 Gy in three fractions or 30 Gy in five fractions

The dose should be prescribed to the highest isodose line encompassing the CTV and be between the 50 and 90% of the maximum dose

*Abbreviation*: *CTV* clinical target volume

Critical organs at risk (OAR) include the optic nerves and optic chiasm. A margin of 1 mm when using mask-based immobilization (no margin when using frame-based immobilization) will be added to each OAR to create each planning OAR volume (PRV). The dose constraints for the optic nerve and chiasm PRVs are Dmax ≤10 Gy in one fraction, ≤20 Gy in three fractions, and ≤ 25 Gy in five fractions; and for Brainstem are Dmax ≤15 Gy in one fraction, ≤23.1 Gy in three fractions, and ≤ 31 Gy in five fractions.

Multiple isocenter, non-isocentric, and non-coplanar beams may be used. Few parameters to be followed while planning and plan evaluation: 95% of the prescribed dose should completely encompass the target (CTV). Dose conformity (for CTV) should be between 1.0 and 2.0 for lesions ≥5 mm in maximum diameter (may be up to 3.0 for lesions < 5 mm in maximum diameter). The minimum and maximum CTV dose, conformity index (defined as the ratio of the prescription isodose volume to the target volume, i.e., CTV), prescription isodose line, maximum point dose to the optic nerves and chiasm, and dose received by 1 cc of the brainstem should be calculated and reported. The isodose distribution for each plan should be submitted in DICOM-RT format. Total PTV volume, normal brain (brain-GTV) volume receiving 12 Gy (V_12 Gy_) will also be recorded.

Patients may receive other medications/treatments as required. Only steroid therapy will be recorded in the case report forms. Cytotoxic chemotherapy should be stopped during SRS. Immunotherapy may be continued during SRS.

### Radiotherapy quality assurance

Efforts will be made to keep treatment interruptions to a minimum when feasible. Treatment interruptions are allowed when clinically required at the discretion of the treating physician, for example to allow for patient recovery after resection or between fractions of fractionated SRS or based on available treatment days given holiday schedules. Radiotherapy compliance criteria are listed in Table 4.

**Table 4:** Radiotherapy compliance criteria

**1.0 Minor deviations**	1.1 < 95% but ≥90% of the prescribed dose completely encompasses the clinical target volume 1.2 > 3 weeks between resection and start of post-operative SRS in the standard arm 1.3 > 1 week between the end of pre-operative SRS and resection in the experimental arm
**2.0 Major deviations**	2.1 > 2 weeks between planning MRI and start of SRS 2.2 Prescribed dose is ≤90% dose required by the study 2.3 < 90% of the prescribed dose completely encompasses the clinical target volume 2.4 Maximum point dose to the optic chiasm is > 10 Gy for treatments delivered in one fraction, > 20 Gy for treatments delivered in three fractions, or > 25 Gy for treatments delivered in five fractions 2.5 Maximum point dose to the brainstem is > 15 Gy for treatments delivered in one fraction, > 3.1 Gy for treatments delivered in three fractions, or > 31 Gy for treatments delivered in five fractions 2.6 > 6 weeks between resection and start of post-operative SRS in the standard arm or > 3 weeks between the end of pre-operative SRS and resection in the experimental arm > 2 weeks to complete a course of fractionated SRS 2.8 Resection is not performed (or cancelled) for any reason

*Abbreviation*: *SRS* stereotactic radiosurgery

### Reporting of adverse events and serious adverse events

This protocol does not contain investigational agents, and adverse events secondary to radiotherapy should be reported in the manner described in Table 5. Common Terminology Criteria for Adverse Events (CTCAE), version 5.0 will be used for adverse event reporting.

**Table 5:** Adverse events reporting

Study Period/Reporting Procedure	Adverse Event Reporting	SAE Reporting
From time patient signs the consent form until 30 days after end of study treatment.	Yes	Yes
Thirty days after the end of study intervention until the last follow up date.	Yes, if assessed as related or possibly related to the treatment/intervention by the Investigator.	Yes, if assessed as related or possibly related to the treatment/intervention by the Investigator.
How to report	Source documents (e.g., study-specific worksheets) and case report form.	SAEs, unexpected, related/possibly related to be reported through a SAE form to the PI (sponsor) with copy to the Investigator-Initiated Trials team within 24 hours REB/Health Canada to be notified within applicable timeframe.

*Abbreviations*: *PI* principal investigator, *REB* research ethics board, *SAE* serious adverse event

### Study assessments

The schedule of the study assessments is listed in Table 6.

**Table 6:** Study assessments

	Screening *(within 7 days of randomization)*^*a*^	End of Protocol Treatment^**b**^ *2 days to 3 weeks*	Follow-up Post-Treatment: 3, 6, 9, 12, 16, 24 months^**c**^ *(± 2 weeks)*
Informed consent	X		
Medical history, handedness	X		
ECOG performance status	X		
Adverse event review	X	X	X
Steroid dose assessment	X	X	X
Urine or serum pregnancy test^d^	X		
Diagnostic MRI scan	X *(within 30 days of randomization)*		X
Quality of life questionnaires	X		X
Neurocognitive testing	X		X
Randomization^e^	X		
Resection with pre- or post-operative SRS		X	

^a^All screening procedures must be completed within 7 days of the treatment start date. Results of tests obtained prior to signing the informed consent conducted as part of the subject’s standard care may be used if performed within 7 days before treatment; ^b^The adverse event review and concomitant/steroid dose assessment will be completed once all study treatments have concluded; ^c^Follow up will commence after the last treatment date. Each follow-up visit has a window of ±2 weeks; ^d^Performed as required as per standard institutional policies; ^e^Randomization will occur after all screening procedures are complete and the patient is found to be eligible for trial

*Abbreviations*: *ECOG* Eastern Cooperative Oncology Group, *SRS* stereotactic radiosurgery

### Participant withdrawal and participant discontinuation

Participants may withdraw from the study at any time at their own request, or they may be withdrawn at any time at the discretion of the investigator or sponsor for safety or behaviour reasons, or the inability of the participant to comply with the protocol required schedule of study visits/procedures.

### Sample size

Group sample size of 44 in standard arm and 44 in the experimental arm (total sample size, 88 patients) achieves 80% power to detect a difference between the group proportions of 0.28. The proportion in the standard arm is assumed to be 0.50 and in the experimental arm is assumed to be 0.78. Two-sided t-tests were used to calculate the sample size. The significance level targeted was 0.05 and the actual significance level achieved by the design is 0.044.

### Statistical analysis

Two types of population will be analyzed:

1. Intent-to-treat population: All patients will be analyzed in the groups to which they were randomized, regardless of whether they received or adhered to the allocated intervention.
2. Per-protocol population: All patients who fulfil eligibility criteria, received, and adhered to treatment assigned, and completed required assessments. For example, patients who had their resection cancelled or not completed will not be analyzed in the per-protocol population analysis.

Interim analysis for futility and efficacy is planned. One interim analysis is planned when 44 events are seen (approximately 50% of total planned events). The interim analysis will be completed and submitted to the Data and Safety Monitoring Board (DSMB) within 60 days of reaching that event. The Pocock beta spending function is used for early stopping for futility and Lan-Demets O’Brien-Flemming alpha spending function is used for early stopping for efficacy.

Descriptive statistics will be used to report baseline characteristics. The mean and standard deviation will be used to describe normally distributed continuous variables; the median and interquartile range will be used to describe non-normally distributed continuous variables. The t-test will be used to compare the continuous variables and the χ2 test will be used to compare categorical variables between arms. Survival will be estimated using the Kaplan-Meier method and compared between arms with two-sided log-rank tests. Hazard ratios will be estimated using the Cox proportional-hazard models adjusted for baseline and stratification variables. Statistical significance will be based on a two-sided α of 0.05. Statistical analyses will be conducted using the current version of SAS at the time of analysis (SAS Institute, Cary, NC).

### Data collection, and data management

An electronic data capture system will be used in this trial. A case report form will be completed for each consented patient. This data will be entered by the trial site into the electronic data capture system.

### Monitoring and data safety

The DSMB will review trial activities like patient recruitment, reported adverse events every six months. The sponsor or designee will monitor the site activity to verify that the rights and well-being of human participants are protected; the reported trial data are accurate, complete, and verifiable from source documents; the conduct of the trial complies with the currently approved protocol/amendment(s), with Good Clinical Practice, and with applicable regulatory requirements(s). It will be ensured that this study is conducted in compliance with the protocol and in agreement with the Declaration of Helsinki.

## Discussion

There is insufficient evidence on the optimal sequence of surgical resection and SRS for brain metastases [23]. While postoperative SRS remains standard of care, retrospective series suggest potential benefits of preoperative SRS. Comparing preoperative SRS and postoperative SRS patients, Patel et al. [15] concluded that there are reductions in SRN and leptomeningeal disease rate in preoperative SRS patients with similar rates of local recurrence, distant brain recurrence and overall survival. Similarly, the PROPS-BM multicenter cohort study confirmed the low rates of LMD and AREs [16].

In the present study, the optimal sequencing of surgery and SRS in the management of patients with resectable brain metastases is being prospectively evaluated. Patients with ≤10 brain metastases will be randomized to postoperative and preoperative SRS arms. Our study allows fractionated SRS in both preoperative and postoperative settings. This will address the issue of larger targets receiving reduced dose with single fraction SRS and will therefore mitigate the risk of local failure with STR as found in the PROPS-BM cohort [16].

While this study allows SRS to be delivered by either LINAC or Gamma-knife, it aims to assess the impact of SRS equipment, total PTV volume, and volume receiving 12 Gy of the normal brain on the clinical outcome, adverse events, quality of life, and neurocognitive outcomes.

In summary, this study is a phase III, multicenter, randomized controlled trial comparing preoperative versus postoperative SRS in patients with resectable brain metastases. It includes patients with up to ten brain metastases, allows fractionated SRS in both the arms, adopts no dose-reduction in the preoperative SRS delivery and assesses clinical as well as quality of life and neurocognitive outcomes.

## Data Availability

Not applicable.

## Abbreviations

ARE: Adverse radiation effect
CTCAE: Common Terminology Criteria for Adverse Events
CTV: Clinical target volume
DSMB: Data and safety monitoring board
EDC: Electronic data capture
GTV: Gross tumor volume
LMD: Leptomeningeal disease
LINAC: Linear accelerator
LR: Local recurrence
OAR: Organ at risk
OS: Overall survival
PRV: Planning organ at risk volume
PTV: Planning target volume
SRN: Symptomatic radiation necrosis
SRS: Stereotactic radiosurgery
STR: Subtotal resection
WBRT: Whole brain radiotherapy
